# RGDC Peptide-Induced Biomimetic Calcium Phosphate Coating Formed on AZ31 Magnesium Alloy

**DOI:** 10.3390/ma10040358

**Published:** 2017-03-28

**Authors:** Lin Cao, Lina Wang, Lingying Fan, Wenjun Xiao, Bingpeng Lin, Yimeng Xu, Jun Liang, Baocheng Cao

**Affiliations:** 1 School of Stomatology, Lanzhou University, Lanzhou 730000, China; caolzw@163.com (L.C.); wangln2014@lzu.edu.cn (L.W.); fanly12@lzu.edu.cn (L.F.); xiaowj14@lzu.edu.cn (W.X.); linbp07@lzu.edu.cn (B.L.); xuym15@lzu.edu.cn (Y.X.); 2 State Key Laboratory of Solid Lubrication, Lanzhou Institute of Chemical Physics, Chinese Academy of Sciences, Lanzhou 730000, China; liangj@ciomp.ac.cn

**Keywords:** biomimetic, calcium phosphate coating, RGDC, magnesium alloy, corrosion resistance, biocompatibility

## Abstract

Magnesium alloys as biodegradable metal implants have received a lot of interest in biomedical applications. However, magnesium alloys have extremely high corrosion rates a in physiological environment, which have limited their application in the orthopedic field. In this study, calcium phosphate compounds (Ca–P) coating was prepared by arginine–glycine–aspartic acid–cysteine (RGDC) peptide-induced mineralization in 1.5 simulated body fluid (SBF) to improve the corrosion resistance and biocompatibility of the AZ31 magnesium alloys. The adhesion of Ca–P coating to the AZ31 substrates was evaluated by a scratch test. Corrosion resistance and cytocompatibility of the Ca–P coating were investigated. The results showed that the RGDC could effectively promote the nucleation and crystallization of the Ca–P coating and the Ca–P coating had poor adhesion to the AZ31 substrates. The corrosion resistance and biocompatibility of the biomimetic Ca–P coating Mg alloys were greatly improved compared with that of the uncoated sample.

## 1. Introduction

Traditional implant materials such as titanium and its alloys, steel, and cobalt–chromium alloys are extensively used in orthopedics [1]. These materials can develop excellent mechanical properties during the tissue healing process. However, they have some problems associated with their applications, such as stress shielding effects and releasing toxic ions into the tissue [2]. These issues can cause the inflammatory response and subsequent loss of bone, which may result in implant loosening [3].

Recently, magnesium and magnesium alloys have attracted much more attention as potential biodegradable implant materials because of their excellent mechanical properties and biocompatibility [4,5]. The specific density and Young’s moduli of Mg are similar to those of human bones, which can reduce the risk of stress shielding effects [6]. However, the Mg alloy extremely high corrosion rate in a physical environment has become one of the major stumbling blocks that limit their development for biomedical applications [7,8]. Many studies for improving the corrosion resistance of magnesium have been attempted, including alloying [9,10], processing [11,12], and protective coating [13,14]. It was known that calcium phosphate compounds (Ca–P) coatings had excellent biocompatibility, osteoconductivity, and non-toxicity in in vivo environment [15]. Several methods for forming the Ca–P coating to improve the corrosion resistance of magnesium alloys, such as chemical deposition, electrodeposition, hydrothermal treatment, and alkali-heat treatment have been reported [16,17]. From among these, the biomimetic method has attracted several focuses because it can offer a simple, low-temperature, non-toxic, and non-line-of-sight process [18]. Moreover, the solution used during the coating process is similar to human plasma to prepare safe, biocompatible coatings.

Many researchers have introduced some active molecules on the surface of magnesium alloys to promote mineralization of Ca–P coatings. Lin et al. [19] prepared a hydroxyapatite (HA) coating by the biomimetic method in a Ca–P solution with the assistance of polydopamine on the surface of AZ31 and confirmed that polydopamine has the ability to induce mineralization of the apatite layer. Gao et al. [20] firstly dipped grapheneoxide (GO) coating on AZ91 and then prepared a HA/GO hybrid coating by a biomimetic method, finding that GO greatly promoted nucleation and crystallization for HA growth. This hybrid coating can effectively improve the corrosion resistance of Mg alloys.

RGD (Arg-Gly-Asp) sequence was identified that it can mediate the attachment of cells to several plasma and extracellular matrixc (ECM) proteins, such as fibronectin, vitronectin, collagen, and laminin. Also, it has the ability to bind with a variety of cells through ligand–receptor interactions. Therefore, researchers have immobilized RGD-containing peptides onto the surface of biomaterials to promote cell attachment [21,22,23]. Cao et al. [24] immobilized RGD peptide on the TiO_2_ nanotubes for investigating its influence on bone mesenchymal stem cells (BMSCs) adhesion and osteogenic gene expression. Secchi et al. [25] confirmed that arginyl-glycyl-aspartyl-serine (RGDS) peptides immobilized on the titanium alloy stimulated bone cell differentiation. In addition to promoting cell attachment, RGD is also rich in carboxyl active functional groups. It has been verified that surface functional groups play an important role in calcium phosphate nucleation [26,27]. However, the studies about whether RGD peptide has influence on the formation of Ca–P coating are rarely reported.

In the present study, arginine–glycine–aspartic acid–cysteine (RGDC) peptide is immobilized onto the surface of AZ31 magnesium alloys through a covalent attachment to induce the formation of Ca–P coating on magnesium alloy in 1.5 simulated body fluid (SBF). The corrosion resistance and biocompatibility of the Ca–P coatings are investigated.

## 2. Results and Discussion

### 2.1. Surface Characterization

**Figure 1:**
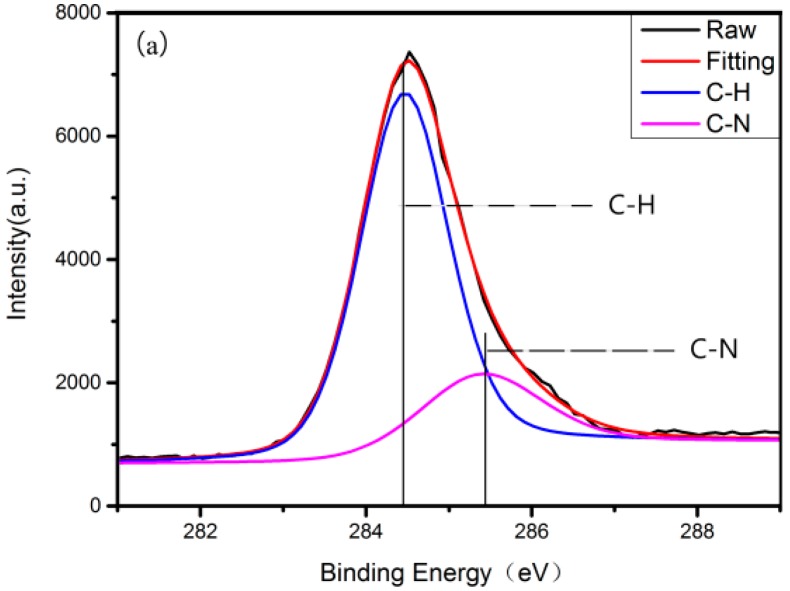

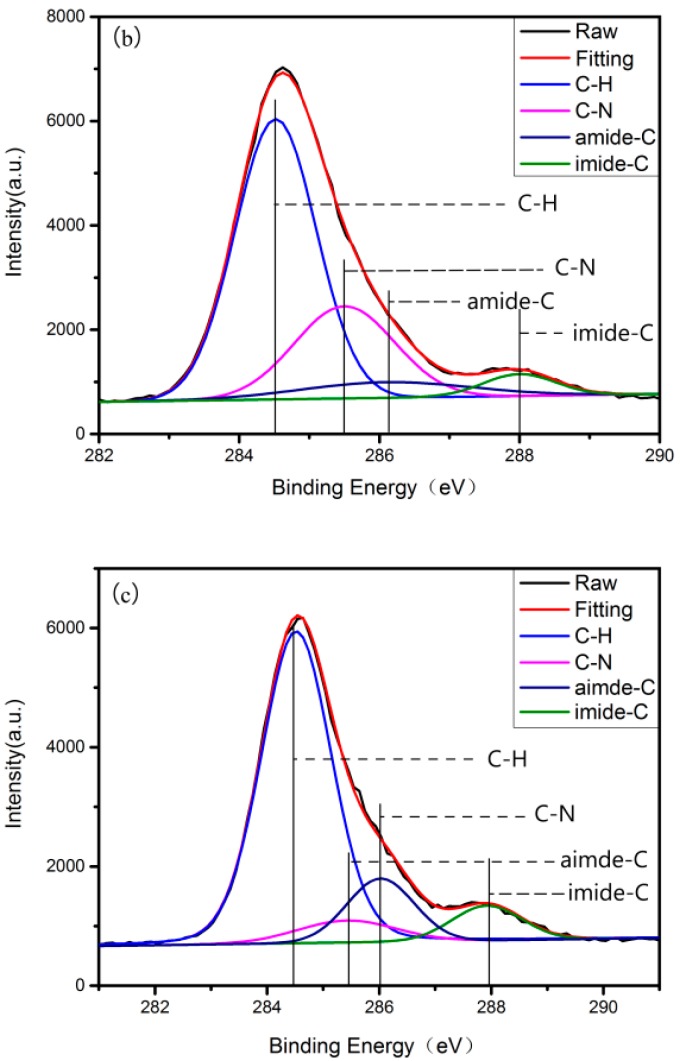
C1s X-ray photoelectron spectroscopy (XPS) spectra obtained at the different steps of the grafting procedure: (**a**) the step of silanization; (**b**) the step of grafting 3-Succinimidyl-3-Mal-eimidoPropionate (SMP); (**c**) the step of arginine–glycine–aspartic acid–cysteine (RGDC) peptide grafting.

After each step of grafting RGD on AZ31, X-ray photoelectron spectroscopy (XPS) was carried out to determine the surface elements. Figure 1 displays the C1s spectrum at each step of grafting RGDC. A-AZ31 principally shows two components, C–H at 284.4 eV and C–N at 285.5 eV (Figure 1a) [28]. After grafting 3-Succinimidyl-3-Mal-eimidoPropionate (SMP), there appear C(=O)N amino groups related to the C1s peak, and amide-C (286.1 eV) and imide-C (288.0 eV) are detected in addition to C–H at 284.4 eV, C–N at 285.5 eV (Figure 1b) [29]. When RGDC peptide was immobilized on the surface of AZ31, the area of amide-C and imide-C increased (Figure 1c).

Table 1 showed that after treating with three-aminopropyltriethoxysilane (APTES), silicon and nitrogen were detected on the surface of AZ31 and N/Si ratio was 3.56/4.1, slightly lower than APTES ratio (1:1), and may be attributed to hydrolysis of the NH_2_ groups [23]. After grafting with SMP, the stoichiometric N/Si ratio is 2:1 and the experimental value gave 5.14:4.29, thus illustrating that SMP grafting effectively takes place without systematic hanging. That may be due to the inadequate reaction of the NH_2_ groups hydrolysis of A-AZ31 with maleimide group in the SMP. There is also a significant increase in nitrogen content, as can be seen from the N/Si ratio found in Table 1.

**Figure 2:**
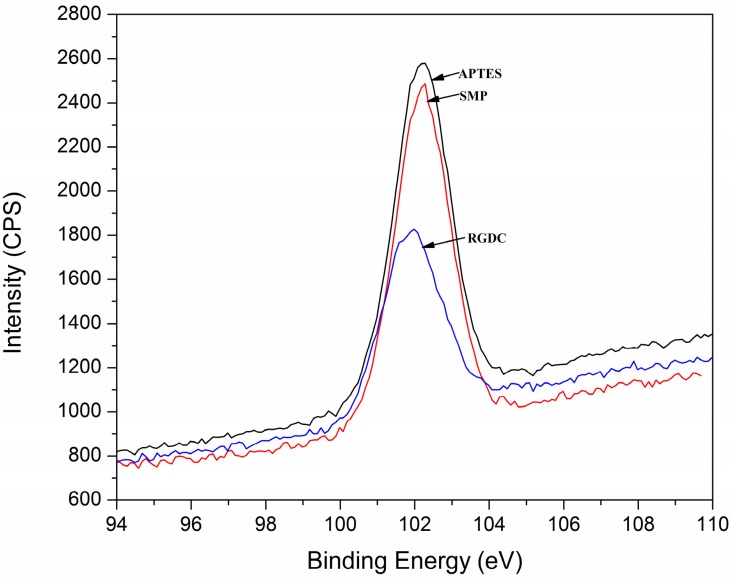
Overlap of XPS Si2p spectra for AZ31 grafting with three-aminopropyltriethoxysilane (APTES), SMP and RGDC.

Figure 2 shows one maximum component at 102.7 eV corresponding to a typical SiO_3_C bonding. Therefore, it can be indicated that the -CH_2_-CH_2_-NH_2_ chains are well grafted on the surface of AZ31. However, no additional modifications were discovered for Si2p spectra.

The RGDC includes a sulfur-containing cysteine residue that can be regarded as a mark for the presence of peptide. When the RGDC has been immobilized, a very small amount of sulfur is detected (Table 1) and Si2p spectra appear at 164 eV and 168.5 eV, suggesting that maleimide groups are covalently bound to the sulfhydryl of RGDC peptide, and RGDC are successfully grafted onto the surface of AZ31.

**Figure 3:**
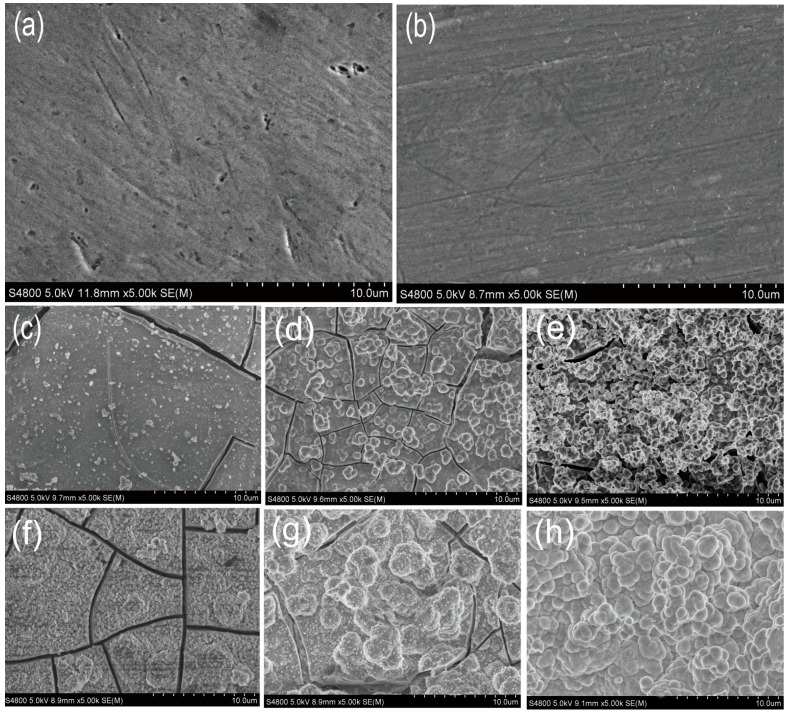
SEM images show the surface morphologies of AZ31 and coated AZ31 alloys: (**a**) AZ31; (**b**) R-AZ31; (**c**–**e**) AZ31, S-AZ31, R-AZ31, after soaking in 1.5 simulated body fluid (SBF) for two days, respectively; (**f**–**h**) AZ31, S-AZ31, R-AZ31, after soaking in 1.5 SBF for seven days, respectively.

Figure 3a,b show the surface morphologies of substrate and R-AZ31. The surface topography of AZ31 was relatively flat with some grinding scratches and pitting (Figure 3a), but the surface became more smooth and uniform after being treated with RGDC, and the pitting disappeared (Figure 3b).

Figure 3c–e show the SEM images of the coatings formed in 1.5 SBF for two days. There are some small irregular particles with a relatively dispersed distribution on the surface of AZ31 and S-AZ31. In addition, a few cracks and defects are observed in the coating (Figure 3c,d). However, a large number of spherical particles with a diameter of about 1–2 µm were deposited on the surface of R-AZ31 and showed less cracks (Figure 3c). From SEM observation, spherical particles increased in number and size and were closely stacked together with longer growth time (seven days) for all the specimens (Figure 3f–h). The surface of AZ31 and S-AZ31 were partially covered by a number of increasing particles, but the cracks are still clearly observed (Figure 3f,g). However, the surface was completely covered by the coating, no cracks and defects were found on the R-AZ31 surface, as seen from Figure 3f. It was obvious that the coating became more uniform and denser with the use of RGDC.

**Figure 4:**
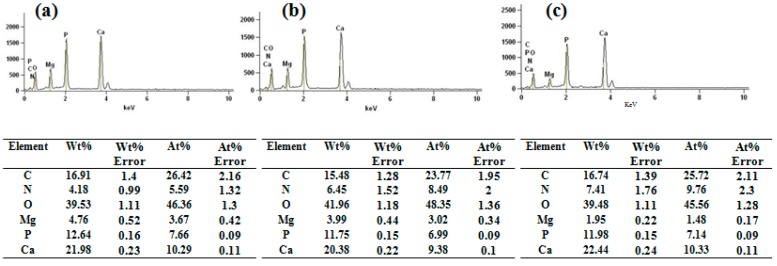
Energy dispersive spectroscopy (EDS) patterns of coated samples with seven days: (**a**) AZ31-HA; (**b**) S-AZ31-HA; (**c**) R-AZ31-HA.

**Figure 5:**
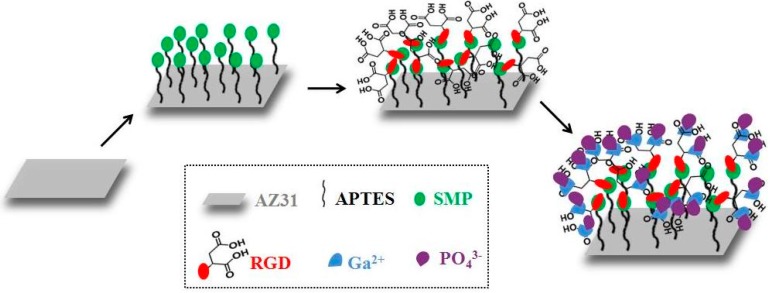
Schematic illustration for the formation mechanism of HA on R-AZ31 Mg alloy.

The energy dispersive spectroscopy (EDS) results indicated that the calcium-to-phosphate ratio was 1.34, 1.34, and 1.47 for AZ31-HA, S-AZ31-HA, and R-AZ31-HA, respectively, as shown in Figure 4. It can be seen that Ca/P ratios of all the coatings were below that of the stoichiometric HAp (1.67), and this was caused in part by the substitutions in the lattice. Calcium phosphate compounds can be substituted with different ions. In the HA lattice, calcium can be replaced by small amounts of magnesium and sodium, and phosphates can be replaced by carbonate ions [30]; moreover, transient and metastable phases in the final product of Ca–P coatings are present, which are generally nonstoichiometric and poorly crystallized [31]. Therefore, based on the SEM and EDS results, these porous spherical structures belonging to calcium-deficient HA could be clearly defined. The growth mechanism of HA on R-AZ31 (Figure 5) is described as the following: in the molecular structure of the RGDC-containing carboxyl group, when R-AZ31 were immersed in 1.5 SBF, a large number of positively charged Ca^2+^ ions in SBF were adsorbed onto the charged RGDC surface. Subsequently, PO_4_^3−^ ions bound to Ca^2+^ ions form a calcium phosphate compound that subjected the mineralization process as a nucleation center. As a result, it formed a uniform dense Ca–P coating on the surface of R-AZ31 [27].

**Figure 6:**
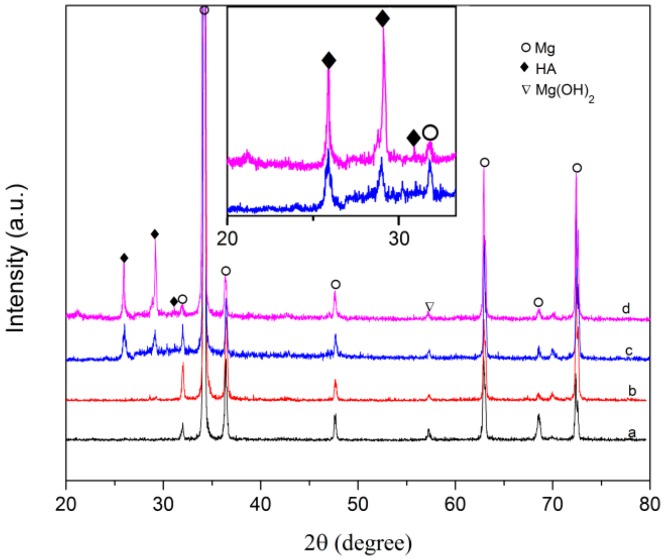
XRD spectra of specimens after immersion in 1.5 SBF for seven days: (**a**) AZ31; (**b**) R-AZ31; (**c**) AZ31-HA; (**d**) R-AZ31-HA.

Figure 6 illustrates X-ray diffraction patterns of the AZ31 substrate with and without calcium phosphate coating. Besides the diffraction peaks of Mg from the AZ31 substrate, new peaks appear for all the coating samples. The peak at 2θ = 26° represents the reflection of (002) plane of HA, and the peak at 2θ = 29.2° is attributed to the reflection of (210) plane of HA phase. In addition, a weak peak was observed at 2θ = 32° in the coating group spectrum, which corresponds to the overlapping of the (211) and (112) diffraction planes and is indicative of a poorly crystalline, bone-like HA. The strongest peak of HA is detected in the R-AZ31 coated group.

**Figure 7:**
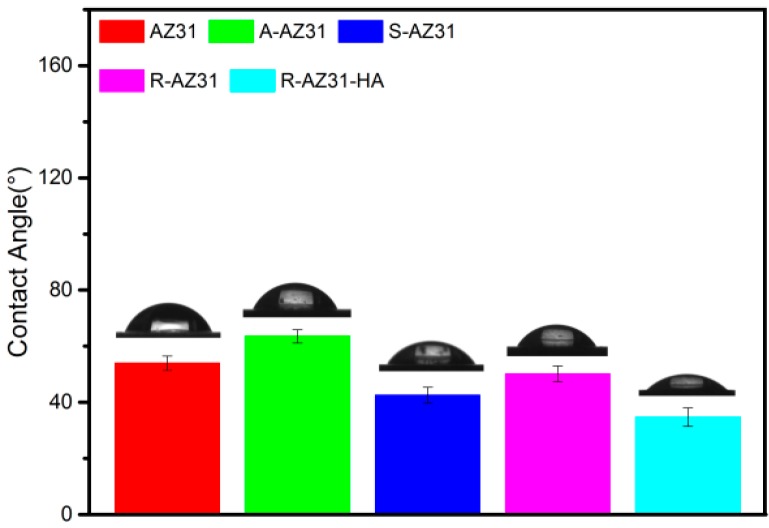
Water contact angles on the samples at different stages.

Figure 7 shows the water contact angles measured from the samples at different stages. The contact angle of AZ31 is 54.6°; after subsequent APTES treatment, it exhibits hydrophobic surface with a contact angle of 63°, and when the SMP and RGDC are grafted, the wettability of the sample considerably increased with a contact angle of 41° and 48°, respectively. However, the contact angle of R-AZ31 reduced after Ca–P coating deposited. The results showed that the Ca–P coating increased hydrophobicity of substrates.

### 2.2. Scratch Adhesion

**Figure 8:**
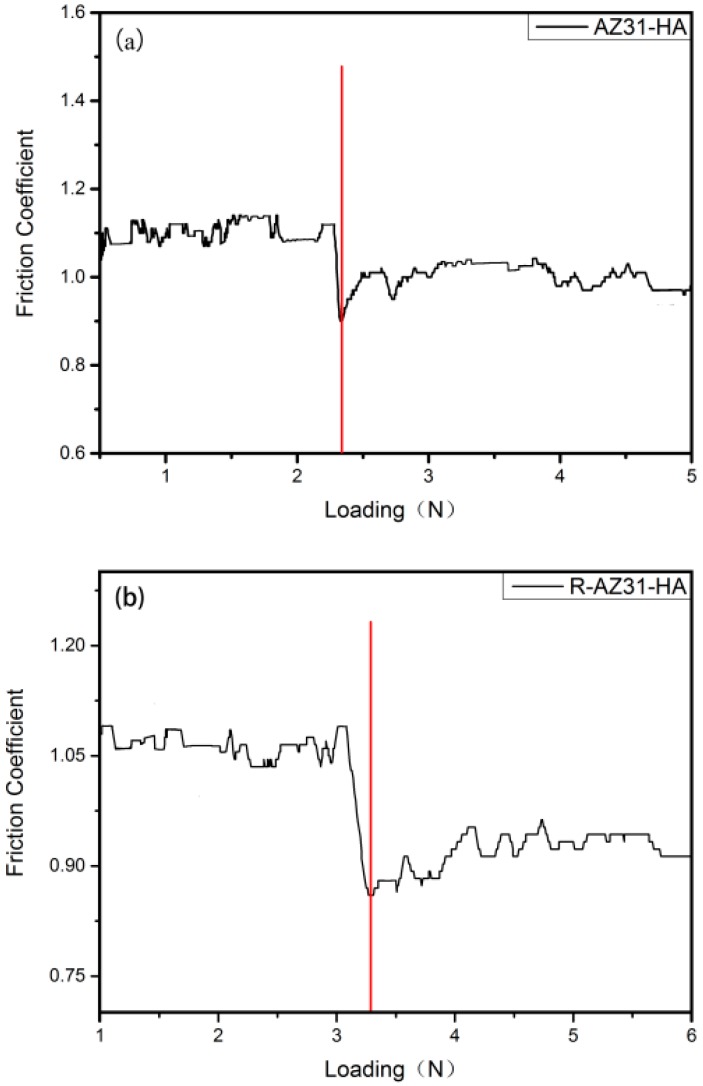
Scratch adhesion test: (**a**) AZ31-HA; (**b**) R-AZ31-HA.

**Figure 9:**
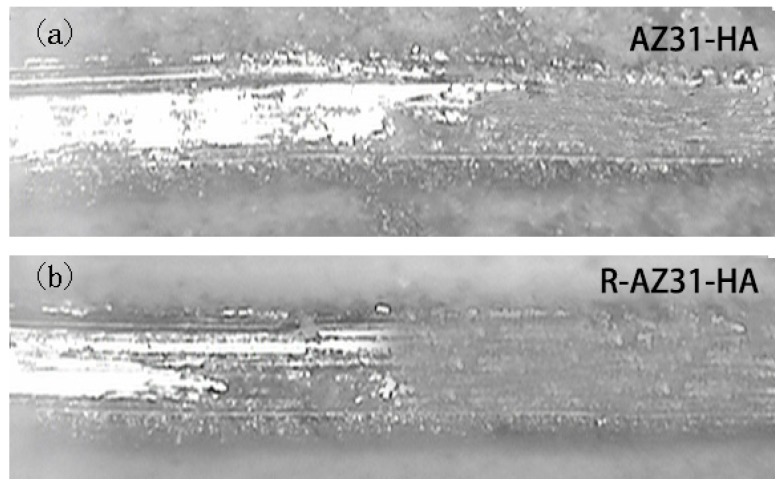
The scratch traces of AZ31-HA and R-AZ31-HA: (**a**) AZ31-HA; (**b**) R-AZ31-HA.

The scratch adhesion test results are shown in Figure 8. To peel off the Ca–P coating from the R-AZ31 surface required about 3.3 N, a slight higher load than AZ31, which was about 2.35 N. The Ca–P coatings were crumbled and smeared out along the path of the pyramid diamond tip before the coatings detached from the substrates (Figure 9). The results confirmed that the Ca–P coatings had a poor strength to the AZ31 substrate.

### 2.3. Corrosion Resistance

**Figure 10:**
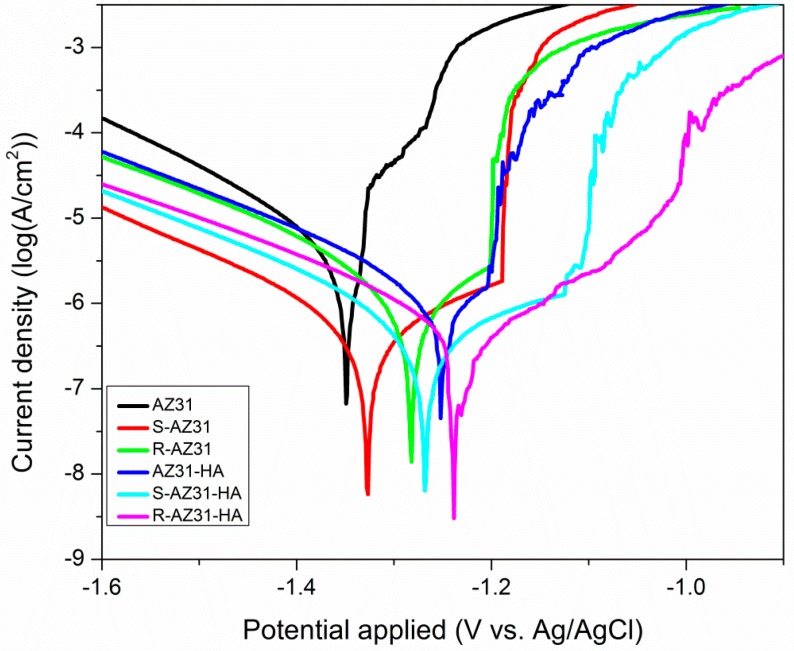
Potentiodynamic polarization curves of the substrate with and without calcium phosphate coating.

Figure 10 presents the potentiodynamic polarization curves of the substrate with and without coating and the corresponding electrochemical parameters are listed in Table 2. It is obvious that AZ31 substrate has the highest *i*_corr_ (1.64 × 10^−5^ A/cm^2^) and the most negative *E*_corr_ (−1.389 V vs. Ag/AgCl) value. The S-AZ31 exhibits more positive *E*_corr_ (−1.339 V vs. Ag/AgCl) and slightly lower *i*_corr_ (8.3 × 10^−6^ A/cm^2^) than that of bare AZ31 substrate, while R-AZ31 shows more positive *E*_corr_ (−1.324 V vs. Ag/AgCl) and slightly lower *i*_corr_ (6.7 × 10^−6^ A/cm^2^) than the S-AZ31. The results showed that the *i*_corr_ decreased and the *E*_corr_ increased with increasing numbers of self-assembled monolayer grafted on the surface of AZ31, which suggests a certain anticorrosion ability of the self-assembled monolayer. The HA coated AZ31 has slightly more positive *E*_corr_ and lower *i*_corr_ compared with R-AZ31, and the *i*_corr_ value of S-AZ31-HA is lower than that of AZ31-HA. This indicates that the corrosion resistance of single calcium phosphate coatings is better than the self-assembled monolayer, while composite coatings have better corrosion resistance than single calcium phosphate coatings [32]. However, after deposition of calcium phosphate coating, the *E*_corr_ of R-AZ31 positively shifted to −1.231 V and its *i*_corr_ decreased to 1.42 × 10^−6^ A/cm^2^, which implies that the composite coatings of R-AZ31-HA have the best effect on improving the corrosion resistance of the substrate in SBF.

The improvement of corrosion resistance for R-AZ31-HA can be attributed to two factors. Firstly, when APTES, SMP and RGDC are grafted on the surface of AZ31, these self-assembled monolayers have a positive effect on providing corrosion protection on AZ31. Secondly, Ca–P coating on the surface of AZ31 is also favorable for improving the corrosion resistance of the substrate. Therefore, composite coating contributes to improving the corrosion resistance of magnesium alloys.

**Figure 11:**
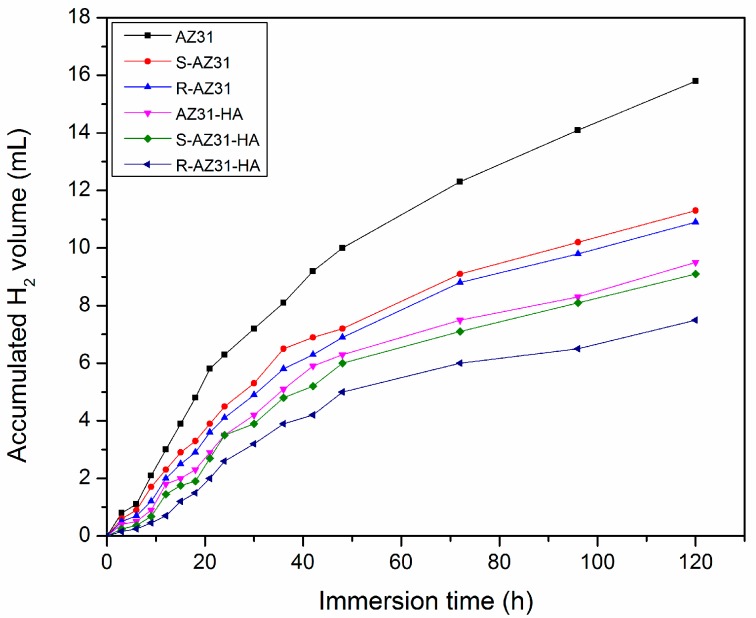
Hydrogen evolution volumes of specimens immersed in SBF for 5 d.

Figure 11 shows the volume variation of hydrogen gas as a function of immersion time in SBF for the samples. For the AZ31, the volume of hydrogen gas increases significantly for the first 40 h immersion and then gradually slows down for the subsequent immersion time to give a total H_2_ evolution volume of 15.8 ml after immersion for five days, which represents the highest corrosion rate. The hydrogen gas evolution rates of S-AZ31 and R-AZ31 are slightly lower than AZ31, with total H_2_ evolution volumes of 11.3 mL and 10.9 mL, respectively, during the same immersion time. The amount of H_2_ has been collected for AZ31-HA during SBF incubation, 9.5 mL/cm^2^ within five days, which is lower than that of R-AZ31 (10.9 mL/cm^2^), but slightly higher than that of S-AZ31-HA (9.1 mL/cm^2^). However, for the R-AZ31-HA, the H_2_ evolution rate slowly increases with the immersion time and only 7.5 mL H_2_ is collected, which shows the lowest corrosion rate among all the specimens, and indicates that the self-assembled monolayer of RGDC and Ca–P coating have corrosion protection to the substrate. These results are consistent with the electrochemical experiment that R-AZ31-HA should have the best corrosion resistance.

### 2.4. Cytotoxicity Evaluation

**Figure 12:**
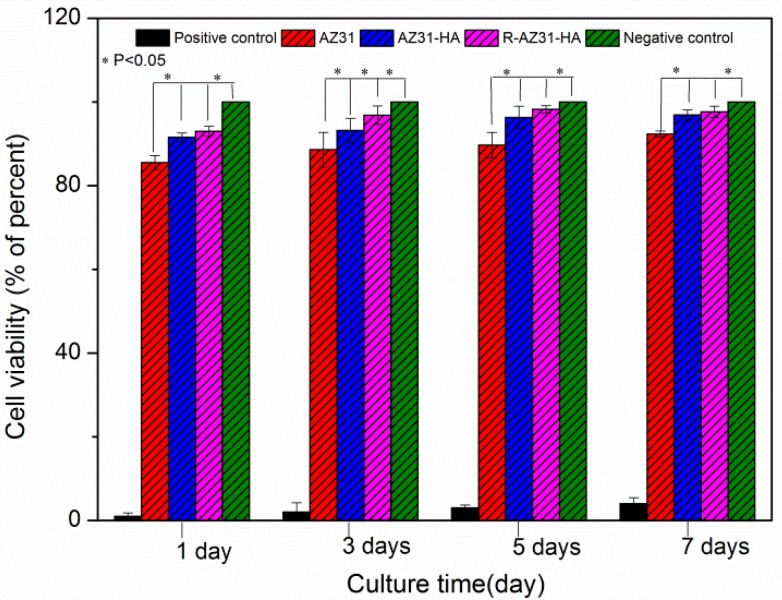
Cell viabilities of L-929 cells cultured in extraction mediums of AZ31, AZ31-HA, R-AZ31-HA.

**Figure 13:**
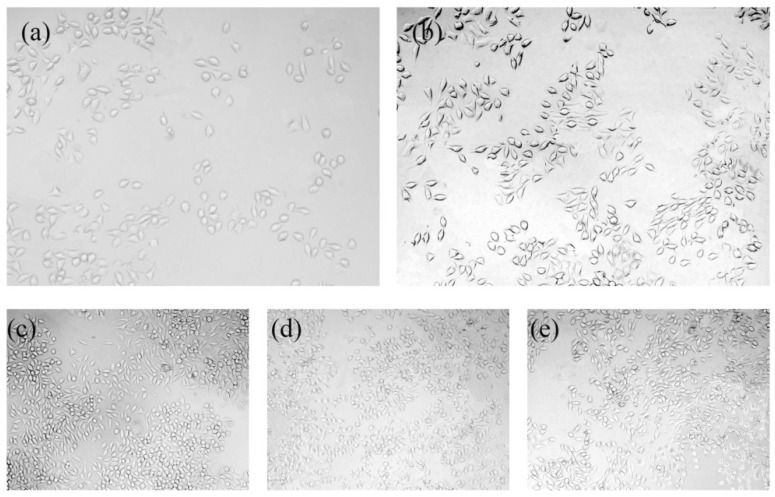
Optical microscopy images (×10) representing the morphologies of L-929 cells cultured for 7 d in (**a**) Positive control group; (**b**) AZ31 extracts; (**c**) AZ31-HA extracts; (**d**) R-AZ31-HA extracts; (**e**) Negative control.

Figure 12 shows the L-929 cell viability cultured with extractions of AZ31, AZ31-HA, R-AZ31-HA for different periods (1, 3, 5 and 7 d). It can be seen that the viability AZ31, AZ31-HA, and R-AZ31-HA are all above 80%, which verifies the non-toxicity of the as-prepared samples. However, the cell viability of the AZ31-HA and R-AZ31-HA groups are better than the AZ31 group at these four times (*p* < 0.05). These results indicate that Ca–P coating on magnesium alloys promotes the cell viability of L-929 cell in vitro. It may be because the dissolution from the Ca–P coating acts as a growth factor in the differentiation of L-929 [33,34]. However, there was no significant difference in cell viability between the AZ31-HA group and R-AZ31-HA group, which indicated that RGDC below the Ca–P coating could not promote the cell proliferation. Figure 13 displays optical microscopy images reflecting the morphologies of the L-929 cells cultured in different sample extracts after 7 d. The cell number of AZ31 extracts was obviously less than AZ31-HA extracts, R-AZ31-HA extracts and negative control. The cell morphology in the uncoated group and the coated group were normal and healthy, similar to that of the cells with elongated spindle morphology incubated in the culture media.

## 3. Materials and Methods

### 3.1. Materials

Three-aminopropyltriethoxysilane (APTES) and 3-Succinimidyl-3-Mal-eimidoPropionate (SMP) were purchased from Sigma-Aldrich, USA. RGDC was synthesized by the biochemical laboratory of Lanzhou University in China. AZ31 Mg alloy with the major elements of approximately 3–3.2 wt % Al, 0.8 wt % Zn and 0.4 wt % Mn was purchased from Hongxing Metal Material Co., Ltd., Dongguan, China.

### 3.2. Sample Preparation

AZ31 were cut into rectangular samples with the size of 10 mm × 10 mm × 1 mm and the surfaces were ground with 2000 grit SiC paper, rinsed ultrasonically with acetone, ethanol, and deionized water for 15 min, respectively, and then air dried. All samples were soaked in 5 M/L NaOH solution for 12 h at 80 °C, aged in water for 4 h at 80 °C, and then air dried.

#### 3.2.1. Silanization with APTES

**Figure 14:**
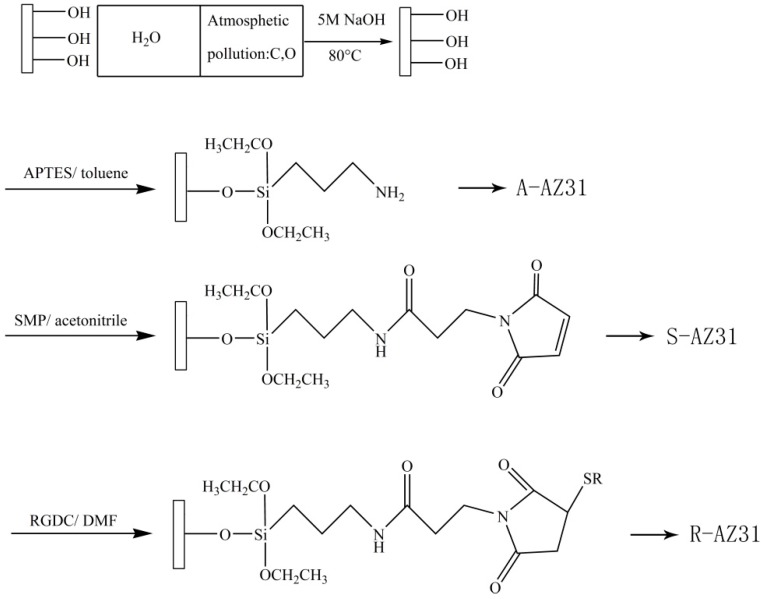
Schematic representation of the chemical grafting group.

RGDC was immobilized by means of a three-step reaction procedure as shown in Figure 14. An aminosilane linker was used to achieve RGDC peptide immobilization on magnesium alloy. Silanization was carried out by immersing the substrates in a solution of APTES in a toluene solution, of which the volume ratio was 1:20, reflux heating for 4 h, and natural cooling for 12 h. Then the samples were rinsed with the toluene solution, ethanol, and deionized water three times, respectively. The samples were labeled as A-AZ31.

#### 3.2.2. Cross-Linking with SMP

Silanized substrates were incubated for 12 h at room temperature with a solution of 20 mL of acetonitrile containing 20 mg of N-succinimidyl-3-Maleimidoproprionate (SMP) and the samples were rinsed with acetonitrile three times to remove unconjugated SMP; then they were rinsed thoroughly with deionized water. The samples were labeled as S-AZ31.

#### 3.2.3. RGDC Peptide Was Immobilized on AZ31

A solution of RGDC was prepared in DMF (N,N-dimethyl-formamide) with the concentration of 1 mg/mL, all the samples were immersed in RGDC solution for 24 h at room temperature, then they were removed and rinsed with DMF and deionized water several times. The samples were labeled as R-AZ31.

### 3.3. Biomimetic Coating

In this study, biomimetic Ca–P coating was prepared in 1.5 SBF. The ion concentrations of SBF and 1.5 SBF are listed in Table 3. The initial pH of 1.5 SBF was adjusted to 7.4 using Tris-HCl buffer. AZ31, S-AZ31 and R-AZ31 samples were immersed in 1.5 SBF for two and seven days, respectively, at 37 °C. Then, the samples were removed, rinsed with deionized water, and air dried.

### 3.4. Surface Characterization

X-ray photoelectron spectroscopy (ESCALAB 250Xi, Thermo Scientific, Waltham, MA, USA) was used at each step of the RGD peptides grafting to determine whether the RGD was successfully immobilized onto the surface of AZ31. The morphology of the coating on the substrate was examined using SEM/EDS analysis with a scanning electron microscope (Hitachi S-4800, Hitachi, Ltd., Tokyo, Japan). The phase compositions of coating were identified by X-ray diffraction (D/MAX-2400, Rigaku Co., Ltd., Tokyo, Japan) with a Cu Kα radiation source at 40 kV and 150 mA. The contact angle of samples at different stages were measured using 4 µL water droplets on a contact angle goniometer (JC2000D1, Shanghai zhongchen digital technic apparatus Co., Ltd., Shanghai, China) at room temperature and the average value was calculated from three measurements conducted on three different samples.

### 3.5. Scratch Adhesion

To evaluate the adhesion force between coating and Mg substrate, a scratch test of substrates was done. A Vickers pyramid diamond tip with a radius of 200 µm is used. At constant speed, the tip is pulled along the surface of the film and as the sample is scanned, the tip is subjected to an increasing load up to a maximum of 5 N. Table 4 shows typical load parameters used for scratch testing. The scratch traces were observed by Optical microscope.

### 3.6. Corrosion Tests

#### 3.6.1. Electrochemical Experiment

The corrosion behaviors of samples were investigated by potentiodynamic polarization curves using a 273A potentiostat on the Autolab PGSTAT302N electrochemical system. The tests were carried out in the simulated body fluid (SBF) at 37 °C. A three-electrode cell was used for electrochemical measurements with an Ag/AgCl electrode (saturated with KCl) as a reference electrode, a platinum plate as a counter electrode, and the biomimetic synthesis specimen as the working electrode. Before the experiment, the samples were stabilized at their open circuit potential (OCP) for 10 min.

#### 3.6.2. Immersion Test

The immersion test was carried out in SBF at 37 °C, and the initial pH was adjusted to 7.4. The hydrogen evolution rate was monitored during the immersion time, and the hydrogen evolution device was the same as described by Shi et al. [35]. After five days, all samples were removed, rinsed with distilled water, and air dried.

### 3.7. Cell Culture

AZ31 samples with and without coating were disinfected by ultraviolet light for 20 min before cell culture. Mouse connective tissue cell line (L-929; Cell Bank at the Chinese Academy of Sciences, Lanzhou, China) were cultured in Dulbecco’s modified Eagle’s medium (DMEM, Gibco), and supplemented with 10% fetal bovine serum (FBS; Sijiqing Hangzhou) and 1% streptomycin and penicillin in a humidified incubator with 5% CO_2_ at 37 °C. The sample extracts were prepared using DMEM serum-free cell culture medium with a surface area/extraction medium ratio of 1 cm^2^/mL, and then incubated in a humidified incubator with 5% CO_2_ at 37 °C for 72 h. The negative control is the DMEM medium and the positive control is the DMEM medium containing 10% dimethylsulfoxide (DMSO; Sigma-Aldrich, St. Louis, MO, USA). L929 were incubated for 24 h in 96-well cell plates at density of 1 × 10^4^ cells/200 µL cell culture medium in each well. Then, the cell culture medium of each well was replaced by 200 µL of extracts for the experimental and control groups. The plates were incubated at 37 °C for 1, 4 and 7 d. At the end of each incubation time, the media was replaced with 10 µL 3-(4,5-dimethylthiazol-2-yl)-2,5-Diphenyltetrazolium bromide (MTT; Sigma-Aldrich, St. Louis, MO, USA), and incubated for 4 h at 37 °C in darkness. Afterwards, 100 μL of a formazan solubilization solution (10% sodium dodecyl sulfate in 0.01 M HCl) was added to each well and the samples were incubated overnight. The optical density (OD) was determined using a microplate reader (Elx 800, Bio-Tek, Winooski, VT, USA) at a wavelength of 570 nm. After incubation for seven days, cell culture photos were obtained using an inverted optical microscope (IX2, Olympus, Tokyo, Japan).

## 4. Conclusions

The present study demonstrated that Ca–P coating was successfully prepared on the AZ31 with the use of RGDC by using a biomimetic method. The contact angle results indicated that RGDC and Ca–P coating increased the wettability of substrates and the scratch adhesion test confirmed that biomimetic Ca–P coating had poor adhesion force to the AZ31 substrate. Potentiodynamic polarization test and hydrogen evolution measurement showed that the formed R-AZ31-HA composite coating significantly improved the anticorrosion ability of magnesium alloy in SBF. The in vitro cellular tests revealed that the coating showed no cytotoxic reaction to L929 and significantly enhanced the cellular responses. These results suggest that biomimetic Ca–P coating on AZ31 with the assistance of RGDC is a promising approach for modifying the biodegradable implant surface.

## Figures and Tables

**Table 1:** XPS atomic composition in percentages of AZ31 at each step of the treatment.

Samples	Mg	C	O	Si	N	S	N/Si
AZ31	21.53 ± 1.0765	37.35 ± 1.8675	39.08 ± 1.954	2.04 ± 0.102			
A-AZ31	16.86 ± 0.843	40.46 ± 2.023	35.02 ± 1.751	4.1 ± 0.205	3.56 ± 0.178		0.87 ± 0.0435
S-AZ31	17.01 ± 0.8505	39.49 ± 1.9745	34.07 ± 1.7035	4.29 ± 0.2145	5.14 ± 0.257		1.19 ± 0.0595
R-AZ31	16.91 ± 0.8455	37.17 ± 1.8585	36.65 ± 1.8325	2.29 ± 0.1145	6.5 ± 0.325	0.48 ± 0.024	2.84 ± 0.142

**Table 2:** The corrosion potential (*E*_corr_) and corrosion current density (*i*_corr_) of the different AZ31 Mg alloy samples.

Samples	*E*_corr_ (V vs. Ag/Cl)	*i*_corr_ (A/cm^2^)
AZ31	−1.389	1.64 × 10^−5^
S-AZ31	−1.339	8.3 × 10^−6^
R-AZ31	−1.324	6.7 × 10^−6^
AZ31-HA	−1.252	6.45 × 10^−6^
S-AZ31-HA	−1.268	4.36 × 10^−6^
R-AZ31-HA	−1.231	1.42 × 10^−6^

**Table 3:** Composition of SBF and 1.5 SBF solutions.

Component	Concentrations	
	SBF(g/L)	1.5 SBF(g/L)
NaCl	8.035	12.053
NaHCO_3_	0.355	0.503
KCl	0.225	0.338
K_2_HPO_4_·3H_2_O	0.231	0.347
MgCl_2_·6H_2_O	0.311	0.467
CaCl_2_	0.292	0.438
Na_2_SO_4_	0.072	0.108

**Table 4:** Scratch adhesion load parameters.

Loading Range	0–5 N
Loading Rate	5 N/min
Scratch Length	5 mm

## References

[1] Witte F., Hort N., Vogt C., Cohen S., Kainer K.U., Willumeit R., Feyerabend F. (2008). Degradable biomaterials based on magnesium corrosion. Curr. Opin. Solid State Mater. Sci..

[2] Navarro M., Michiardi A., Castano O., Planell J.A. (2008). Biomaterials in orthopaedics. J. R. Soc. Interface R. Soc..

[3] Lutz R., Srour S., Nonhoff J., Weisel T., Damien C.J., Schlegel K.A. (2010). Biofunctionalization of titanium implants with a biomimetic active peptide (P-15) promotes early osseointegration. Clin. Oral Implant. Res..

[4] Staiger M.P., Pietak A.M., Huadmai J., Dias G. (2006). Magnesium and its alloys as orthopedic biomaterials: A review. Biomaterials.

[5] Feng A., Han Y. (2010). The microstructure, mechanical and corrosion properties of calcium polyphosphate reinforced ZK60A magnesium alloy composites. J. Alloys Compd..

[6] Fekry A.M., Tammam R.H. (2012). Electrochemical Behavior of Magnesium Alloys as Biodegradable Materials in Phosphate Buffer Saline Solution. Int. J. Electrochem. Sci..

[7] Song G. (2007). Control of biodegradation of biocompatable magnesium alloys. Corros. Sci..

[8] Hornberger H., Virtanen S., Boccaccini A.R. (2012). Biomedical coatings on magnesium alloys—A review. Acta Biomater..

[9] Cipriano A.F., Zhao T., Johnson I., Guan R.G., Garcia S., Liu H. (2013). In vitro degradation of four magnesium-zinc-strontium alloys and their cytocompatibility with human embryonic stem cells. J. Mater. Sci. Mater. Med..

[10] Du H., Wei Z., Liu X., Zhang E. (2011). Effects of Zn on the microstructure, mechanical property and bio-corrosion property of Mg–3Ca alloys for biomedical application. Mater. Chem. Phys..

[11] Peng Q., Zhao H., Li H., Ma N., Tian Y. (2012). High Pressure Solidification: An Effective Approach to Improve the Corrosion Properties of Mg-Y Based Implants. Int. J. Electrochem. Sci..

[12] Zhang X., Yuan G., Mao L., Niu J., Fu P., Ding W. (2012). Effects of extrusion and heat treatment on the mechanical properties and biocorrosion behaviors of a Mg-Nd-Zn-Zr alloy. J. Mech. Behav. Biomed. Mater..

[13] Li M., Cheng Y., Zheng Y.F., Zhang X., Xi T.F., Wei S.C. (2012). Surface characteristics and corrosion behaviour of WE43 magnesium alloy coated by SiC film. Appl. Surf. Sci..

[14] Yang J., Cui F., Lee I.S. (2011). Surface modifications of magnesium alloys for biomedical applications. Ann. Biomed. Eng..

[15] Gan J., Tan L., Yang K., Hu Z., Zhang Q., Fan X., Li Y., Li W. (2013). Bioactive Ca–P coating with self-sealing structure on pure magnesium. J. Mater. Sci. Mater. Med..

[16] Shadanbaz S., Dias G.J. (2012). Calcium phosphate coatings on magnesium alloys for biomedical applications: A review. Acta Biomater..

[17] Li K., Wang B., Yan B., Lu W. (2012). Preparing Ca-P coating on biodegradable magnesium alloy by hydrothermal method: In vitro degradation behavior. Chin. Sci. Bull..

[18] Ribeiro A.A., Balestra R.M., Rocha M.N., Peripolli S.B., Andrade M.C., Pereira L.C., Oliveira M.V. (2013). Dense and porous titanium substrates with a biomimetic calcium phosphate coating. Appl. Surf. Sci..

[19] Lin B., Zhong M., Zheng C., Cao L., Wang D., Wang L., Liang J., Cao B. (2015). Preparation and characterization of dopamine-induced biomimetic hydroxyapatite coatings on the AZ31 magnesium alloy. Surf. Coat. Technol..

[20] Gao F., Xu C., Hu H., Wang Q., Gao Y., Chen H., Guo Q., Chen D., Eder D. (2015). Biomimetic synthesis and characterization of hydroxyapatite/graphene oxide hybrid coating on Mg alloy with enhanced corrosion resistance. Mater. Lett..

[21] Chua P.H., Neoh K.G., Kang E.T., Wang W. (2008). Surface functionalization of titanium with hyaluronic acid/chitosan polyelectrolyte multilayers and RGD for promoting osteoblast functions and inhibiting bacterial adhesion. Biomaterials.

[22] Nguyen M.N., Lebarbe T., Zouani O.F., Pichavant L., Durrieu M.C., Heroguez V. (2012). Impact of RGD nanopatterns grafted onto titanium on osteoblastic cell adhesion. Biomacromolecules.

[23] Abdul Kafi M., El-Said W.A., Kim T.H., Choi J.W. (2012). Cell adhesion, spreading, and proliferation on surface functionalized with RGD nanopillar arrays. Biomaterials.

[24] Cao X., Yu W.Q., Qiu J., Zhao Y.F., Zhang Y.L., Zhang F.Q. (2012). RGD peptide immobilized on TiO_2_ nanotubes for increased bone marrow stromal cells adhesion and osteogenic gene expression. J. Mater. Sci. Mater. Med..

[25] Secchi A.G., Grigoriou V., Shapiro I.M., Cavalcanti-Adam E.A., Composto R.J., Ducheyne P., Adams C.S. (2007). RGDS peptides immobilized on titanium alloy stimulate bone cell attachment, differentiation and confer resistance to apoptosis. J. Biomed. Mater. Res. A.

[26] Mao C., Li H., Cui Q., Feng Q., Wang H., Ma C. (1998). Oriented growth of hydroxyapatite on (0001) textured titanium with functionalized self-assembled silane monolayer as template. J. Mater. Chem..

[27] Liu Q., Ding J., Mante F.K., Wunder S.L., Baran G.R. (2002). The role of surface functional groups in calcium phosphate nucleation on titanium foil: A self-assembled monolayer technique. Biomaterials.

[28] Bartouilh de Taillac L., Porté-Durrieu M.C., Labrugère C., Bareille R., Amédée J., Baquey C. (2004). Grafting of RGD peptides to cellulose to enhance human osteoprogenitor cells adhesion and proliferation. Compos. Sci. Technol..

[29] Porte-Durrieu M.C., Guillemot F., Pallu S., Labrugere C., Brouillaud B., Bareille R., Amedee J., Barthe N., Dard M., Baquey C. (2004). Cyclo-(DfKRG) peptide grafting onto Ti-6Al-4V: Physical characterization and interest towards human osteoprogenitor cells adhesion. Biomaterials.

[30] Shadanbaz S., Walker J., Staiger M.P., Dias G.J., Pietak A. (2013). Growth of calcium phosphates on magnesium substrates for corrosion control in biomedical applications via immersion techniques. J. Biomed. Mater. Res. B Appl. Biomater..

[31] Gómez-Morales J., Iafisco M., Delgado-López J.M., Sarda S., Drouet C. (2013). Progress on the preparation of nanocrystalline apatites and surface characterization: Overview of fundamental and applied aspects. Prog. Cryst. Growth Charact. Mater..

[32] Liu P., Pan X., Yang W., Cai K., Chen Y. (2012). Improved anticorrosion of magnesium alloy via layer-by-layer self-assembly technique combined with micro-arc oxidation. Mater. Lett..

[33] Abdal-hay A., Amna T., Lim J.K. (2013). Biocorrosion and osteoconductivity of PCL/nHAp composite porous film-based coating of magnesium alloy. Solid State Sci..

[34] Laurencin D., Almora-Barrios N., de Leeuw N.H., Gervais C., Bonhomme C., Mauri F., Chrzanowski W., Knowles J.C., Newport R.J., Wong A. (2011). Magnesium incorporation into hydroxyapatite. Biomaterials.

[35] Shi Z., Atrens A. (2011). An innovative specimen configuration for the study of Mg corrosion. Corros. Sci..

